# 2747. Aztreonam-Avibactam Susceptibility Testing Program for Metallo-β-Lactamase-Producing Enterobacterales collected through the Antimicrobial Resistance Laboratory Network, March 2019 to April 2023

**DOI:** 10.1093/ofid/ofad500.2358

**Published:** 2023-11-27

**Authors:** Amelia Bhatnagar, Porscha Bumpus-White, Sarah Sabour, Janine Bodnar, Albert Burks, Arlene Chen, Bradley Craft, Christine Jacobsen, Maria Karlsson, Michael Marmerow, David Lonsway, Lindsay Neff, Tyler Maruca, Elizabeth Nazarian, Zachary Perry, Alessandro Rossi, Paula S Vagnone, Michael Tran, Ann M Valley, Allison C Brown

**Affiliations:** Eagle Medical Services, Atlanta, GA; Centers for Disease Control and Prevention, Atlanta, GA; Centers for Disease Control and Prevention, Atlanta, GA; New York State Dept of Health, Albany, New York; Tennessee Department of Health, Nashville, Tennessee; Maryland Department of Health, Baltimore, Maryland; Minnesota Department of Health, St. Paul, Minnesota; New York State Dept of Health, Albany, New York; Centers for Disease Control and Prevention, Atlanta, GA; Wisconsin State Laboratory of Hygiene, Madison, Wisconsin; CDC, Atlanta, GA; Utah Public Health Laboratory, Salt Lake City, Utah; Maryland Department of Health, Baltimore, Maryland; Wadsworth, Albany, NY; Division of Laboratory Services, TN Department of Health, Nashville, Tennessee; Utah Public Health Laboratory, Salt Lake City, Utah; MN EIP, Minneapolis, Minnesota; Washington State Department of Health, Shoreline, Washington; WISCONSIN STATE LAB OF HYGIENE, Madison, WI; Centers for Disease Control and Prevention, Atlanta, GA

## Abstract

**Background:**

Metallo-β-lactamase-producing Enterobacterales (MBL-E) are a major public health concern as they cause infections with few effective treatment options. Aztreonam-avibactam (AZA) is a β-lactam/β-lactamase inhibitor combination agent in the drug development pipeline that is active against MBL-E. Though clinical studies are ongoing, the effective components can be administered by combining two FDA-approved drugs: aztreonam and ceftazidime-avibactam. In 2019, CDC initiated a program in the Antimicrobial Resistance Laboratory Network (AR Lab Network), the Expanded Antimicrobial Susceptibility Testing Program for Hard-to-Treat Infections, to provide antimicrobial susceptibility testing (AST) of MBL-E for AZA. We provide a summary of the program’s results.

**Methods:**

Seven AR Lab Network laboratories validated the use of a digital dispenser to create custom broth microdilution panels for AZA AST. Testing requests must be for clinical decision-making purposes. Isolates must be an Enterobacterales and have laboratory results positive for an MBL gene (i.e., *bla*_NDM_, *bla*_VIM_, or *bla*_IMP_), or be not susceptible to all β-lactams tested. AZA testing results for confirmed MBL-E were reported back to submitters within 3 working days from receipt of the isolate and shared with CDC.

**Results:**

From March 2019 through April 2023, AZA AST was performed for 250 isolates: 103 *Escherichia coli*, 86 *Klebsiella pneumoniae*, 46 *Enterobacter cloacae* complex, 8 *K. oxytoca*, 2 *Providencia rettgeri*, 1 *K. aerogenes*, 1 *Morganella morganii*, 1 *Proteus mirabilis*, 1 *E. hormaechei*, and 1 *Citrobacter amalonaticus*. Carbapenemase genes detected were: 206 *bla*_NDM_, 24 *bla*_NDM_/*bla*_OXA-48-like_, 14 *bla*_KPC_/*bla*_NDM_, 2 *bla*_IMP_, 2 *bla*_KPC_/*bla*_IMP_, and 2 *bla*_NDM_/*bla*_IMP_. All isolates were resistant to ceftazidime-avibactam; 227/250 (90.8%) were not susceptible to aztreonam (≥8 µg/mL). For isolates not susceptible to aztreonam, the addition of avibactam resulted in a median 128-fold MIC reduction of aztreonam. The MIC range of AZA was ≤0.03/4 - >64/4 µg/mL. Overall, the MIC_50_ and MIC_90_ of AZA were 0.5/4 µg/mL and 8/4 µg/mL, respectively; the MIC_50_ and MIC_90_ for *E. coli* were higher than other species, 4/4 µg/mL and 16/4 µg/mL.

**Conclusion:**

Our data suggest that AZA has potent *in vitro* activity against MBL-E collected in the United States.

**Disclosures:**

**All Authors**: No reported disclosures

